# Comparing video-assisted and verbal explanations in Japanese hospital dentistry: patient evaluations in third molar extraction and medical-dental collaborative care

**DOI:** 10.1186/s12911-026-03643-6

**Published:** 2026-06-20

**Authors:** Kousuke Matsumoto, Izumi Mitani, Takako Tsutsui, Shunichi Fujimoto, Mai Osugi, Reina Wakabayashi, Makiko Tanakura, Shungo Furudoi, Masaya Akashi

**Affiliations:** 1 Department of Oral and Maxillofacial Surgery, Japan Community Health Care Organization, Kobe Central Hospital, Kobe, Japan; 2 Department of Oral and Maxillofacial Surgery, Kobe University Graduate School of Medicine, Kobe, Japan; 3 Graduate Course of Health and Social Services, Saitama Prefectural University, Koshigaya, Japan; 4 Department of Anesthesiology, Japan Community Health Care Organization, Kobe Central Hospital, Kobe, Japan; 5 Department of Oral and Maxillofacial Surgery, Hironokougen Hospital, Miki, Japan; 6 Department of Oral and Maxillofacial Surgery, Konan Medical Center, Kobe, Japan

**Keywords:** Video-assisted explanation, Informed consent, Third molar extraction, Multidisciplinary care, Perioperative oral management, Patient education

## Abstract

**Background:**

Video-assisted explanations may support standardized patient information delivery, but their role in hospital dentistry remains insufficiently evaluated. This study aimed to compare patient evaluations of video-assisted and verbal explanations in three common explanation scenarios in a Japanese hospital dental department.

**Methods:**

An anonymous self-administered questionnaire survey was conducted among patients receiving explanations about preoperative consent for third molar extraction, postoperative home care after tooth extraction, or perioperative oral management for inpatients scheduled for surgery under general anesthesia. A total of 297 questionnaire responses were analyzed. Explanation clarity, appropriateness of explanation length, and changes in fear or anxiety were compared between verbal explanation and video-assisted explanation groups. Associations between participant characteristics and questionnaire outcomes were also explored.

**Results:**

No statistically significant differences were observed between the verbal explanation and video-assisted explanation groups in any of the three explanation scenarios for explanation clarity, appropriateness of explanation length, or fear/anxiety scores. No statistically significant differences were observed between male and female participants for any questionnaire item. Age was not significantly associated with explanation clarity or appropriateness of explanation length, whereas a weak negative correlation was observed between age and fear/anxiety scores.

**Conclusion:**

In a hospital dental setting, patient evaluations did not differ significantly between video-assisted and verbal explanations across three common explanation scenarios. Rather than replacing verbal communication, video-assisted explanations may serve as an additional option for patient information delivery.

**Supplementary Information:**

The online version contains supplementary material available at https://doi.org/10.1186/s12911-026-03643-6.

## Background

Informed consent (IC) is a crucial process that ensures patients understand the purpose, methods, and risks of medical procedures and can make autonomous decisions. However, conventional verbal or written explanations often fail to achieve complete patient understanding, particularly owing to the complexity of medical terminology [1, 2]. The use of visual aids such as videos has gained attention as a way to improve this process, especially in routine medical practice. These tools are expected to standardize explanations, improve patient satisfaction, and reduce healthcare providers’ burden [3–8].

In Japan, where healthcare systems face growing human resources constraints, developing more efficient and patient-centered explanatory methods has become crucial. Similar challenges exist in hospital dental departments, which require standardized yet frequently repeated explanations. Our hospital originally developed video explanations in 2020 in response to the coronavirus disease 2019 (COVID-19) pandemic, aiming to reduce face-to-face explanation time while maintaining communication quality.

Three explanation topics are common in the dental department of this hospital: (1) preoperative consent for third molar extraction, (2) postoperative home care instructions following tooth extraction, and (3) perioperative oral management for inpatients scheduled for surgery under general anesthesia. While these explanations can be standardized, variations in individual patient comprehension and emotional responses require careful consideration of delivery methods [9].

This study aimed to compare patient evaluations of video-assisted and verbal explanations in three common hospital dental explanation scenarios, focusing on explanation clarity, appropriateness of explanation length, and changes in fear or anxiety.

## Methods

An anonymous self-administered questionnaire survey was conducted between July 2020 and March 2021 in the Department of Oral and Maxillofacial Surgery at Kobe Central Hospital. Eligible participants were patients receiving one of three explanations: (1) preoperative consent for third molar extraction (pre-extraction), (2) postoperative home care instructions following tooth extraction (post-extraction), or (3) perioperative oral management (POM).

Participants were divided into two groups: those receiving standard verbal explanations alone and those receiving video-assisted explanations. Allocation was not randomized and was based on routine clinical practice. The choice of explanation method was determined by the attending clinician according to individual patient circumstances and clinical judgment. Printed pamphlets covering the same information were used in both groups. The video-assisted group received a standardized video in addition to the pamphlet, whereas the verbal explanation group received explanations based on the pamphlet alone. Individualized verbal explanations were provided in both groups when clinically necessary. All videos were developed in-house by hospital staff.

Each completed questionnaire was treated as an independent response. Because the survey was anonymous, it was not possible to determine whether the same patient contributed more than one questionnaire across different explanation scenarios.

The evaluation included three items: 1) explanation clarity, rated from 0 to 10, with higher scores indicating greater clarity; 2) appropriateness of explanation length, rated from 0 to 10, with higher scores indicating greater appropriateness; and 3) changes in fear or anxiety, rated from − 10 to + 10, where negative values indicate reduced fear or anxiety, positive values indicate increased fear or anxiety, and 0 indicates no change. The questionnaire consisting of these items was newly developed for this study, and an English version is provided as a supplementary file 1. The proportion of participants scoring ≥ 8 for items 1) and 2), and the proportion reporting increased anxiety (> 0) for item 3), were compared using chi-square tests. Scores of ≥ 8 were used descriptively to indicate highly positive evaluations and were not intended as a validated cutoff value. Mann–Whitney U tests were used to compare questionnaire scores between the verbal explanation and video-assisted explanation groups. Associations between participant characteristics (age and sex) and questionnaire outcomes were also explored. Questionnaire scores were compared between male and female participants using the Mann–Whitney U test, and associations between age and questionnaire scores were evaluated using Spearman’s rank correlation coefficients. Statistical significance was set at *p* < 0.05.

The video durations were as follows: pre-extraction: 351s (covering purpose, procedure, complications, and sequelae); post-extraction: 217s (covering bleeding control, hygiene, bathing, physical activity, and alcohol intake); POM: 189s (explaining the role of dental care in preventing pneumonia and tooth damage during intubation for medically complex surgical inpatients).

All analyses were conducted using the SPSS software (Excel Bell Curve). Before questionnaire completion, participants received an explanation of the study purpose and questionnaire content, and informed consent was obtained. This study was approved by the hospital ethics committee (Approval No. 208).

## Results

A total of 325 questionnaires were collected; 28 were excluded owing to incomplete responses, yielding 297 for analysis. Because questionnaires were collected anonymously, it was not possible to determine whether the same patient completed more than one questionnaire across different explanation scenarios. Therefore, questionnaire responses rather than unique patients were used as the unit of analysis. The mean age of the participants was 50.4 years (109 males, 188 females). Explanation topic distribution was: pre-extraction (105 patients: 47 verbal/58 video), post-extraction (99 patients: 44/55), and POM (93 patients: 46/47) (Table 1). Mean ages by group were 38 years (pre-extraction), approximately 50 years (post-extraction), and approximately 65 years (POM), with the POM group containing more older patients. Female predominance was observed in the pre- and post-extraction groups.

**Table 1:** Patient characteristics by explanation topic

Explanation Topic	Verbal Explanation Only (*n* = 137)	Video-Assisted Explanation (*n* = 160)
**Sex (male/female)**: n	56 / 81	53 /107
**Age (mean ± SD**,** years)**	52.3 ± 21.5	49.2 ± 20.9
**Pre-extraction explanation**: n	47	58
Sex (male/female)	17 / 30	16 / 42
Age (mean ± SD, years)	39.9 ± 17.3	36.8 ± 15.6
**Post-extraction explanation;** n	44	55
Sex (male/female)	16 / 28	15 / 40
Age (mean ± SD, years)	52.4 ± 23.2	47.6 ± 21.3
**Perioperative oral management**: n	46	47
Sex (male/female)	23 / 23	22 / 25
Age (mean ± SD, years)	64.8 ± 15.8	66.2 ± 13.3

Table 2 presents the comparison results. No statistically significant differences were observed between the verbal explanation and video-assisted explanation groups in any of the three explanation scenarios (pre-extraction, post-extraction, and POM), for explanation clarity, appropriateness of explanation length, or fear/anxiety scores.

**Table 2:** Comparison of patient evaluation scores between verbal-only and video-assisted groups, by explanation topic

Explanation Topic	Verbal Only	Video-Assisted	*p*-value
**Pre-extraction explanation**	47	58	
explanation clarity (score ≥ 8), n (%)	46 (97.9)	55 (94.8)	0.42
Mean score ± SD	9.81 ± 0.82	9.24 ± 1.79	0.26
Appropriate explanation length (score ≥ 8), n (%)	42 (89.4)	54 (93.1)	0.5
Mean score ± SD	9.32 ± 1.89	9.29 ± 1.97	0.55
Increase in fear/anxiety (score > 0), n (%)	10 (21.3)	17 (29.3)	0.35
Mean score ± SD	−1.03 ± 5.28	0.27 ± 3.77	0.29
**Post-extraction home care explanation**	44	55	
explanation clarity (score ≥ 8), n (%)	43 (97.7)	50 (90.9)	0.16
Mean score ± SD	9.59 ± 1.05	9.44 ± 1.22	0.59
Appropriate explanation length (score ≥ 8), n (%)	39 (88.6)	48 (87.3)	0.84
Mean score ± SD	9.07 ± 2.37	8.89 ± 2.41	0.68
Increase in fear/anxiety (score > 0), n (%)	4 (9.1)	5 (9.1)	1
Mean score ± SD	−3.43 ± 4.96	−2.44 ± 5.49	0.46
**Perioperative oral management**	46	47	
explanation clarity (score ≥ 8), n (%)	41 (89.1)	43 (91.5)	0.7
Mean score ± SD	9.26 ± 1.34	9.29 ± 1.51	0.63
Appropriate explanation length (score ≥ 8), n (%)	36 (78.3)	39 (83.0)	0.56
Mean score ± SD	8.35 ± 3.26	8.85 ± 2.68	0.32
Increase in fear/anxiety (score > 0), n (%)	4 (8.7)	1 (2.1)	0.16
Mean score ± SD	−1.74 ± 4.24	−2.09 ± 3.83	0.77

Notes: Chi-square tests were used for categorical data (participants with scores ≥ 8 for explanation clarity or appropriateness of explanation length, and participants reporting increased fear/anxiety [score > 0]). The Mann–Whitney U test was used to compare mean questionnaire scores between groups. Fear/anxiety scores ranged from − 10 to + 10, where negative values indicated reduced fear/anxiety after the explanation, positive values indicated increased fear/anxiety, and 0 indicated no change

Associations of participant sex and age with questionnaire outcomes are presented in Table 3. No statistically significant differences were observed between male and female participants for any questionnaire item. Age was not significantly associated with explanation clarity or appropriateness of explanation length, whereas a weak negative correlation was observed between age and fear/anxiety scores (ρ = −0.166, *p* = 0.004).

**Table 3:** Associations of sex and age with questionnaire outcomes

variable	explanation clarity	Appropriate explanation length	Fear/anxiety score
Sex: male (*n* = 109)	9.45 ± 1.18	9.17 ± 2.21	-1.25 ± 4.34
Sex: female(*n* = 188)	9.42 ± 1.45	8.86 ± 2.61	-1.91 ± 5.01
p-value	0.458	0.423	0.140
Age correlation, Spearman’s ρ	-0.011	0.055	-0.166
p-value	0.86	0.35	0.004*

Notes: Questionnaire scores according to sex are presented as mean ± standard deviation (SD). Comparisons between male and female participants were performed using the Mann–Whitney U test. Associations between age and questionnaire outcomes were evaluated using Spearman’s rank correlation coefficients (ρ). Statistical significance was defined as *p* < 0.05. *p < 0.05

## Discussion

Although empirical evaluation of digital tools for informed consent (IC) remains limited, previous research has suggested that incorporating multimedia into conventional IC can improve patient understanding and knowledge retention [8]. These findings support the two primary purposes of IC: protecting patient autonomy and reducing medical risk through effective communication [10].

In this study, no statistically significant differences were observed between video-assisted and verbal explanations across three common explanation scenarios in a hospital dental setting. This finding should not be interpreted as evidence that video-assisted explanations are superior to verbal explanations. Rather, it suggests that video-assisted explanations may be positioned as one possible explanatory approach in hospital dentistry.

One possible explanation for this finding is that institution-specific factors, such as patients’ expectations regarding verbal interaction and the communication culture of the hospital, may influence how explanations are received. Elwyn et al. emphasized that shared decision-making involves not only the provision of information but also meaningful dialogue between healthcare providers and patients [11]. In clinical settings where interpersonal communication is highly valued, verbal explanations may provide trust and reassurance that cannot be fully replicated by video alone. Therefore, the absence of statistically significant differences between video-assisted and verbal explanations in the present study may partly reflect the influence of such dialogic elements on patient evaluations. However, these factors were not directly assessed in this study and should be examined in future research.

Patient satisfaction with medical explanations has been reported to vary according to demographic characteristics such as age, highlighting the importance of tailoring communication formats to patient backgrounds [12]. Many patients receiving perioperative oral management (POM) are older adults, and their cognitive and health literacy characteristics may influence how they process and retain medical information. Previous studies have shown that patients with limited literacy, including older adults and those unfamiliar with medical terminology, may have difficulty fully understanding conventional verbal explanations and participating in autonomous decision-making unless communication is appropriately adapted [13–15]. In the present study, age was not associated with explanation clarity or perceived appropriateness of explanation length, whereas a weak negative correlation was observed between age and fear/anxiety scores. This suggests that older participants tended to report greater reductions in fear or anxiety after the explanations; however, the correlation was weak and should be interpreted cautiously.

The approximately 10-year difference in mean age between the pre-extraction and post-extraction groups may reflect differences in the underlying patient populations. Pre-extraction explanations were provided mainly to patients undergoing third molar extraction, who were generally younger. In contrast, post-extraction explanations were provided not only after third molar extraction but also after extraction of other teeth, including extractions related to periodontal disease and other conditions, which likely increased the proportion of older patients. Therefore, age-related findings in this study should be interpreted with consideration of differences in explanation content and patient populations.

POM has been shown to reduce postoperative complications, such as pneumonia and accidental tooth loss during intubation under general anesthesia, through preoperative oral hygiene management [16]. Perioperative oral function management was also introduced into Japan’s public health insurance system through the 2012 revision of the medical fee schedule [17]. Unlike third molar extraction, which is invasive and often associated with heightened anxiety, POM generally involves oral hygiene guidance as part of systemic medical treatment. Although its purpose may not always be intuitively understood by patients, it is generally not a procedure that directly induces anxiety.

At the same time, POM requires collaboration between medical and dental professionals and often involves complex clinical judgments and communication due to the high prevalence of multimorbidity and elevated nursing care needs [18–21]. In such settings, standardized videos may provide a means of delivering general explanatory content at a consistent level. This may allow dental professionals to devote more attention to individualized clinical judgments, such as evaluating mobile teeth or managing perioperative risks. In addition, although not directly demonstrated by the present findings, video explanations may also be applicable beyond POM to other complex care settings involving multiple professionals or transitions between hospitals and long-term care facilities. In such situations, where explanations may be provided by several healthcare professionals, maintaining consistency of information is important. Standardized videos may serve as a shared explanatory tool to support collaboration across professionals and institutions. However, these effects were not directly evaluated in this study and require further investigation.

In contrast, third molar extraction is a routine procedure in hospital dentistry but carries risks such as nerve injury, requiring patient-specific explanations [22]. Previous studies have reported that videos may influence patient anxiety in the context of third molar surgery [23–26]. In the present study, however, no statistically significant differences in fear/anxiety scores were observed between the video-assisted and verbal explanation groups. Additional analyses also showed no association between sex and any questionnaire item. Although previous research has reported that female patients tend to show higher anxiety levels, such differences were not observed in the present study [27]. This discrepancy may reflect differences in study populations or clinical settings.

Although third molar extraction has been widely studied, few studies have evaluated the use of video explanations for postoperative home care instructions. Patients often undergo tooth extraction on an outpatient basis and may experience bleeding, pain, or swelling at home, which can lead to confusion or anxiety. Because even preoperative information may be poorly retained, visual reinforcement may be useful for postoperative instructions [28]. In the present study, no statistically significant differences were observed between video-assisted and verbal explanations in this setting.

Recently, interactive and personalized multimedia tools have emerged as promising approaches in IC [29]. Future explanatory models should consider both social and individual factors. At the same time, clinician involvement remains essential for addressing patient-specific concerns and needs. In this context, video-assisted explanations should not be viewed as a replacement for verbal communication. Instead, they may be regarded as one option for supporting clear, consistent, and repeatable delivery of patient information.

Standardized videos can deliver a defined amount of information within a short period of time and may therefore be useful as an explanatory tool. These videos have been used continuously in routine clinical practice at the study institution. However, the effects of video use on workflow efficiency, optimal use of human resources, and reduction in explanation time were not directly evaluated in this study and should be examined in future research.

This study has several limitations. First, the evaluation was based on self-administered patient questionnaires and may therefore have been influenced by recall bias or individual expectations. In addition, the questionnaire used in this study was newly developed for this study and consisted of three brief single-item measures. Its validity and reproducibility have not been sufficiently examined; therefore, the findings should be interpreted with caution.

Second, allocation to the verbal explanation or video-assisted explanation group was based on routine clinical practice rather than randomization, and selection bias cannot be excluded. In addition, in the video-assisted group, verbal explanations were provided after video viewing, making it difficult to isolate the independent effect of the video itself.

Third, because the questionnaire was anonymous, it was not possible to completely exclude the possibility that the same patient completed questionnaires in more than one explanation scenario. In particular, patients who received both pre-extraction and post-extraction explanations may have completed questionnaires after each explanation. Therefore, the unit of analysis in this study should be interpreted as questionnaire responses after each explanation rather than individual patients.

Finally, this study was conducted at a single institution, mainly among patients referred from local dental clinics. This referral system and the institution-specific clinical environment may have influenced patients’ expectations, prior experiences, and perceptions of the explanations. These limitations should be considered when interpreting the findings.

## Conclusion

In a hospital dental setting, patient evaluations did not differ significantly between video-assisted and verbal explanations across three common explanation scenarios. Rather than replacing verbal communication, video-assisted explanations may serve as an additional option for patient information delivery.

## Supplementary Information

Below is the link to the electronic supplementary material.

Supplementary Material 1

## Data Availability

The datasets generated and/or analyzed in the current study are available from the corresponding author upon reasonable request.

## Abbreviations

POM: Perioperative oral management
JCHO: Japan Community Health Care Organization
