# Supplementary material for: Comparing video-assisted and verbal explanations in Japanese hospital dentistry: patient evaluations in third molar extraction and medical-dental collaborative care
Source: BMC Med Inform Decis Mak. 2026 Jun 20;26:337. doi: 10.1186/s12911-026-03643-6 (PMC13548520; doi:10.1186/s12911-026-03643-6)
Supplement: Supplementary file 1 [file 12911_2026_3643_MOESM1_ESM.pdf]

supplementary file 1: Patient questionnaire used in this study

Please help us by completing this questionnaire regarding the explanation you received today (for one of the following: preoperative explanation for third molar extraction,/postoperative home care instructions following tooth extraction/ perioperative oral management).

For each item below, please select the point on the scale that best reflects your opinion.

1. Clarity of the explanation

Difficult to understand Neutral Easy to understand

0 5 10

2. Appropriateness of the explanation length

Too long / Too short Just right

0 5 10

3. Change in fear or anxiety after the explanation

Decreased No change Increased

-10 0 +10
